# Dr. Lawrence A. Highland

**Published:** 1910-05

**Authors:** 


Dr. Lawrence A. Highland, of Buffalo, died at the contagious diseases hospital in this city, April 2, 1910, from scarlet fever contracted while attending a patient, aged 30 years. He graduated at the University of Buffalo, Medical Department, in 1903.

